# Delayed Diagnosis of X-Linked Adrenal Hypoplasia Congenita in a Boy with a Novel *NR0B1* Variant: A Case Report

**DOI:** 10.3390/children12111469

**Published:** 2025-10-31

**Authors:** Shin-Hee Kim, Kyoung Soon Cho

**Affiliations:** 1 Department of Pediatrics, Incheon St. Mary’s Hospital, College of Medicine, The Catholic University of Korea, Seoul 06591, Republic of Korea; kshped@catholic.ac.kr; 2 Department of Pediatrics, Bucheon St. Mary’s Hospital, College of Medicine, The Catholic University of Korea, Seoul 06591, Republic of Korea

**Keywords:** adrenal hypoplasia congenita, NR0B1, DAX-1, adrenal insufficiency, hypogonadotropic hypogonadism

## Abstract

**Highlights:**

**What are the main findings?**

- We report a pediatric male patient with adrenal insufficiency and hypogonadotropic hypogonadism caused by a novel *NR0B1* variant (c.833_835dup p.(Leu278dup)).
- The case highlights delayed diagnosis despite neonatal symptoms, ultimately leading to adrenal crisis and hypogonadotropic hypogonadism.

**What are the implications of the main findings?**

- *NR0B1* mutations should be considered in boys presenting with adrenal insufficiency and hypogonadotropic hypogonadism, even without a family history.
- Early genetic testing is essential for timely diagnosis, optimal treatment, and genetic counseling, helping to prevent life-threatening complications.

**Abstract:**

NR0B1 (DAX-1) is an orphan nuclear receptor essential for the development and regulation of the adrenal glands and gonads. Pathogenic variants in *NR0B1* cause X-linked adrenal hypoplasia congenita (AHC), which typically presents with adrenal insufficiency and hypogonadotropic hypogonadism (HH) in boys. Delayed diagnosis during adolescence is uncommon but, when it occurs, can lead to preventable adrenal crisis, underscoring the need for early recognition of atypical presentations. We describe a 14-year-old boy who presented with adrenal insufficiency and delayed puberty. Genetic testing revealed a novel hemizygous in-frame duplication variant of *NR0B1* (NM_000475.4:c.833_835dup p.(Leu278dup)). This variant has not been previously reported in association with X-linked AHC. The patient received hydrocortisone (10–12 mg/m^2^/day) and fludrocortisone (0.1 mg/day) as replacement therapy for adrenal insufficiency, along with testosterone supplementation (100–240 mg/day) to induce pubertal progression. Plasma ACTH levels gradually decreased from 10,175 pg/mL at diagnosis to 215 pg/mL during follow-up, accompanied by clinical improvement in skin pigmentation and pubertal development. This case underscores the importance of *NR0B1* genetic testing in children with adrenal insufficiency and HH. Early recognition and genetic confirmation are critical for appropriate management and genetic counseling. Identification of novel variants expands the *NR0B1* mutational spectrum and enhances our understanding of genotype–phenotype correlations in X-linked AHC.

## 1. Introduction

Adrenal hypoplasia congenita (AHC) is a rare inherited adrenal gland development disorder that predominantly affects males [1]. Most cases are X-linked and associated with *DAX-1* variants, although rare autosomal recessive forms have been described [2]. Deletions or mutations of *DAX-1* (dosage-sensitive sex reversal, adrenal hypoplasia critical region on chromosome X, gene 1), also known as *NR0B1* (nuclear receptor subfamily 0, group B, member 1), cause the X-linked form of AHC [3]. The DAX-1 transcription factor plays an important role in the development and function of the adrenal gland, gonads, and pituitary gland, and disruption of this gene can lead to a variety of clinical phenotypes [4]. Males typically present with primary adrenal insufficiency or isolated mineralocorticoid deficiency, hypogonadotropic hypogonadism (HH), and impaired spermatogenesis in infancy or early childhood, but other forms of AHC with delayed onset or mild symptoms have been described [5].

Over 200 pathogenic variants of *NR0B1* have been identified in patients with X-linked AHC, and the complex relationship between genotype and clinical phenotype can contribute to diagnostic delays. Therefore, early diagnosis of affected individuals is critical to prevent life-threatening adrenal insufficiency, and the current treatment approach consists of a glucocorticoid and mineralocorticoid replacement. In addition, treating HH in affected boys requires gonadotropins and/or sexual hormone replacement during puberty and throughout life [6]. This report describes a pediatric male patient with adrenal insufficiency and HH, who was diagnosed with X-linked AHC caused by a novel hemizygous *NR0B1* variant.

## 2. Subject and Methods

### 2.1. Case

The patient was born at 40 weeks’ gestation, weighing 2.7 kg, via spontaneous vaginal delivery. He was the only child of a healthy, nonconsanguineous Korean couple. During the neonatal period, he had poor feeding and recurrent vomiting. Tthese symptoms, along with biochemical abnormalities, including hyponatremia and metabolic acidosis, resolved with fluid therapy. Physical examination revealed normal male external genitalia, with bilaterally descended testes and no evidence of micropenis. Hormone levels, including adrenocorticotropic hormone (ACTH) and 17-OH progesterone, were within the normal range, and no further evaluation was performed. During childhood, he was hospitalized several times for recurrent vomiting with mild hyponatremia and underwent appendectomy at the age of seven, without any evidence of adrenal crisis during or after surgery.

The patient was admitted to Bucheon St. Mary’s Hospital at 14 years of age due to recurrent vomiting and abdominal pain. The patient’s height and weight were 161.8 cm and 68 kg, respectively. Physical examination revealed hyperpigmentation on the lips and over the entire body. He was in a prepubertal stage with a testicular volume of 2 mL, no pubic hair, and a small penis, and his bone age was delayed at 12 years. Laboratory testing showed hyponatremia (Na 122 mEq/L; ref 135–145) and K 5.1 mEq/L (ref 3.5–5.5). At 8:00 a.m., ACTH was markedly elevated (10,175 pg/mL; ref 10–60), cortisol was low (7.5 µg/dL; ref 6.7–22.6), and 17-hydroxyprogesterone was low (0.19 ng/mL; ref 0.6–3.4), consistent with primary adrenal insufficiency. Testosterone was <10 ng/dL (ref 650–810), with low LH (<1 mIU/L; ref 2–9) and FSH (1.1 mIU/L; ref 2–9). A gonadotropin-releasing hormone (GnRH) stimulation showed a blunted pituitary response (baseline LH 0.01, FSH 0.7; 60 min LH 2.1, FSH 1.1 mIU/L). Adrenal computed tomography (CT) demonstrated bilateral adrenal hypoplasia. Pituitary magnetic resonance imaging (MRI) revealed no abnormalities. No evidence of infectious, metabolic, or infiltrative disease was identified.

Glucocorticoid replacement was initiated with oral hydrocortisone at approximately 10 mg/m^2^/day, administered in three divided doses, and was titrated to 12 mg/m^2^/day during adolescence. Fludrocortisone 0.1 mg daily was continued throughout follow-up, with electrolytes and plasma renin remaining stable. At 15 years of age, testosterone enanthate was initiated at 50 mg intramuscularly every four weeks. The dose was progressively escalated to 240 mg every four weeks by age 17 to support pubertal progression and maintain virilization. Under this regimen, the patient developed normal secondary sexual characteristics without complications. The patient’s treatment and follow-up schedule are summarized in Table 1.

Due to the presence of adrenal insufficiency with HH, we suspected that the patient had AHC with an underlying variant in the *NR0B1.* A sample of the patient’s peripheral blood was collected for molecular analysis after informed consent was provided.

### 2.2. Molecular Analysis and Results

For DNA extraction and analysis, blood was collected in 2 mL ethylenediaminetetra-acetic acid (EDTA)-treated tubes. All coding exons and flanking intronic regions of the *NR0B1* were amplified by polymerase chain reaction and sequenced directly. Genetic analysis identified a hemizygous in-frame duplication in *NR0B1* (NM_000475.4:c.833_835dup (p.Leu278dup)). This novel variant has not been reported in public databases, including ClinVar and gnomAD, and is currently classified as a variant of uncertain significance (VUS) per ACMG/AMP guidelines [7]. Segregation analysis could not be performed because both parents declined genetic testing, limiting confirmation of co-segregation and precluding application of PP1 evidence under ACMG/AMP guideline [7].

## 3. Discussion

We report a case of a male pediatric patient with AHC and HH who carried a novel *NR0B1* in-frame duplication variant (NM_000475.4:c.833_835dup p.(Leu278dup)). The initial presentation of pathogenic *NR0B1* often is associated with a combination of mineral and glucocorticoid deficiency in the neonatal period, and the patient can exhibit delayed puberty [8,9]. AHC typically manifests within the first two months of life, though symptoms may emerge during childhood or adolescence [10]. In addition, adult-onset AHC has been described in the recent literature [11,12].

In this case, vomiting, poor feeding, and hyponatremia occurred in the neonatal period, but hormone levels such as ACTH and 17-OH progesterone were normal at that time, and symptoms recovered after fluid treatment. Due to the observed recovery, no further examinations were conducted. At age 14, due to clinical presentation and laboratory assessment results, he was suspected to have primary adrenal insufficiency with HH. Genetic analysis revealed a novel *NR0B1* in-frame duplication variant (NM_000475.4:c.833_835dup p.(Leu278dup)).

In male neonates with suspected primary adrenal insufficiency, once 21-hydroxylase deficiency has been excluded by 17-OH progesterone screening, NR0B1-related X-linked adrenal hypoplasia congenita should be considered. This condition results from pathogenic variants in the *NR0B1* gene, which plays a critical role in adrenal and gonadal development. *NR0B1* is located in the short arm of the X chromosome and is expressed in the adrenal cortex, gonads, hypothalamus, and pituitary gland [13]. Functional studies indicate that NR0B1 (DAX-1) represses gene transcription partly by inhibiting the activity of steroidogenic factor-1 (SF-1), which is involved in sex differentiation, and has also been proposed to act antagonistically to SRY [14,15]. The p.Leu278dup variant in *NR0B1* identified in our patient lies within the N-terminal portion of the ligand-binding domain (LBD; amino acids 260–470), which is essential for SF-1 interaction and the transcriptional repression of adrenal and gonadal targets. Variants affecting neighboring residues within this region, such as p.Leu293Pro and p.Ser315Phe, have been reported to destabilize local folding and/or weaken SF-1 binding, resulting in loss of repressor function [3,13]. Accordingly, it is plausible that the in-frame duplication at Leu278 perturbs the local conformation of the LBD and partially impairs DAX-1 repressor activity, which is consistent with the classical presentation of adrenal insufficiency and HH in our patient.

Since the first description of the *NR0B1* variant as a cause of AHC in 1994, several new variants have been discovered of which deletions, nonsense variants, and frameshift variants of the carboxyl-terminal were most common [8]. However, the clinical heterogeneity of NR0B1-related AHC remains diagnostically challenging. A recent study showed that even though two patients had the same variant, there were some differences in clinical features such as age onset [11]. Some patients have an adrenal crisis within a few days after birth, while others do not develop adrenal insufficiency until adulthood [16]. This reflects a combination of influences including genetic (modifier genes, and variability in expressivity and penetrance) and environmental factors (intercurrent illnesses and other stressors [17]. Other possible explanations for these variations is the differences in salvage mechanisms for persistence of fetal adrenal glands, which suggests that other factors, including epigenetics and protein modifications, are involved in the process [13]. Table 2 summarizes cases of *NR0B1* variants with AHC with HH and the characteristics described in the recent literature.

A previous study reported 117 male children with unexplained adrenal insufficiency, 58% of whom were confirmed to have the *NR0B1* variants. Even in the abscence of a family history, 45% of the cases were found to have the *NR0B1* variants [18]. Therefore, based on clinical presentation when more common causes are ruled out, testing for the *NR0B1* variants is an important consideration for patients with adrenal insufficiency.

**Table 2:** AHC cases in the recent literature including age at diagnosis, clinical manifestation, and genetic analysis.

Reference	Age at Diagnosis	Age at Onset, Days	Clinical Manifestation	HH	Family History	ACTH (pg/mL)	Genetic Analysis
This case	14 years	5 days	Vomiting, abdominal pain	Presence	Absence	10,175	c.833_835dup p.(Leu278dup)
[11]	1 years	1 year	Vomiting, hypoglycemia	Presence	Presence	–	c.604delT p.(C202Afs*62)
[11]	2 months	3 days	Salt wasting, hyperpigmentation	–	Presence	1188	c.604delT p.(C202Afs*62)
[12]	28 years	9 years	Failure to thrive, skin hyperpigmentation	Presence	Absence	388	c.154G>T p.(Glu52Term)
[8]	8 months	18 days	Salt wasting, feeding intolerance	Presence	Presence	91.4	c.848_849delinsCC p.(Gln283Pro)
[19]	24 years	36 days	Salt wasting	Presence	Presence	2000	c.543delA p.(Glu52Term)
[20]	51 years	4 years	Failure to thrive, vomiting, hyperpigmentation	Presence	Presence	–	c.571_574delGGGC
[20]	43 years	9 years	Failure to thrive, vomiting, hyperpigmentation	Presence	Presence	–	c.571_574delGGGC
[21]	40 years	30 years	Weight loss, salt craving, hyperpigmentation	Presence	Presence	1151	p.Tyr378Cys
[21]	64 years	58 years	Hypotension, nausea, hyponatremia, hyperpigmentation	Presence	Presence	1012	p.Tyr378Cys
[21]	36 years	30 years	Dizziness, weight loss, hyperpigmentation, fatigue	Absence	Presence	>1250	p.Tyr378Cys

ACTH: adrenocorticotropic hormone; HH: hypogonadotropic hypogonadism.

We report the case of male adolescent who presented at age 14 with adrenal insufficiency with HH at the age of 14 and was diagnosed with AHC caused by a novel *NR0B1* variants. It is important to diagnose AHC early and accurately to allow proper management as well as facilitate adequate genetic counseling for the family. The present study suggests that the combination of primary adrenal insufficiency and HH at any age should raise the suspicion of *NR0B1* variants, which requires immediate genetic testing. In addition, X-linked AHC and HH should be considered even in the absence of a family history.

In conclusion, this case contributes to the expanding phenotypic and clinical spectrum of NR0B1-related conditions, that AHC may present during adolescence in association with HH. Recognition of such atypical, late-onset manifestations is of clinical relevance, as early and adequate hormone replacement therapy is essential to prevent adrenal crisis and to ensure appropriate pubertal development. Further evidence, such as confirmed de novo occurrence and disease-relevant functional studies, is required to more firmly establish this novel variant as causative.

## Figures and Tables

**Table 1:** Summary of auxological parameters, laboratory findings, and treatments during a 5-year follow-up.

	Chronological Age (Years)					
Variables	14	15	16	17	18	19
Height (cm)	161	166	170.9	172.8	173	173
Weight (kg)	70.0	78.0	78.5	82.0	88.7	83.4
Bone age (years)	12	15	17	18	not assessed	not assessed
Pubertal stage ^a^	1	3	4	5	5	5
Testicular volume (mL) ^b^	2/2	8/8	10/10	12/12	14/14	16/16
ACTH (pg/mL)	10,175	9550	4932	2250	3232	215
Cortisol (ng/mL) ^c^	2.3	1.38	0.46	0.49	0.68	0.53
17-OH progesterone (ng/mL)	0.19	0.06	<0.04	<0.04	<0.03	<0.03
DHEA-S (ng/mL)	7.72	<2.64	<2.64	3.22	8.21	4.95
Testosterone (ng/dL)	<10	185	156.6	180.53	65.65	missing
Aldosterone (ng/dL)	<1	<1	1.94	1.33	2.17	3.07
Plasma renin activity (ng/mL/h)	10.08	2.64	3.23	6.73	>20	>20
Treatment						
Hydrocortisone (mg/m^2^/day)	10	12	12	12	12	12
Fludrocortisone (mg/day)	0.1	0.1	0.1	0.1	0.1	0.1
Testosterone (mg) ^d^		100–150	200	240	240	240

^a^ Pubertal stage was evaluated according to the Tanner scale for genital and pubic hair development. ^b^ Testicular size was measured using a Prader orchidometer and recorded in milliliters (mL). ^c^ Morning blood samples were obtained at 8 a.m., before the first hydrocortisone dose of the day. ^d^ Administrated intramuscularly every four weeks. ACTH; adrenocorticotropic hormone; DHEA-S; dehydroepiandrosterone sulfate.

## Data Availability

The original contributions presented in the study are included in the article; further inquiries can be directed to the corresponding authors.
